# Efficacy and Safety of Combination Therapy With Vaginal and Urethral Erbium-Doped Yttrium-Aluminum-Garnet (Er:YAG) Laser for Overactive Bladder With Urinary Incontinence

**DOI:** 10.7759/cureus.62363

**Published:** 2024-06-14

**Authors:** Nobuo Okui, Machiko A Okui

**Affiliations:** 1 Urogynecology, Yokosuka Urogynecology and Urology Clinic, Kanagawa, JPN; 2 Urology, Yokosuka Urogynecology and Urology Clinic, Kanagawa, JPN

**Keywords:** er:yag laser, vaginal health index score, nocturia, daytime frequency, voided volume, international consultation on incontinence questionnaire-short form, overactive bladder symptom score, overactive bladder, vaginal and urethral erbium:yttrium aluminum garnet laser

## Abstract

Objective: This retrospective cohort study with propensity score (PS) matching aimed to evaluate the efficacy and safety of a combination therapy with vaginal and urethral erbium:yttrium aluminum garnet laser (VEL+UEL) (SP Dynamis; Fotona d.o.o., Ljubljana, Slovenia) in the treatment of overactive bladder with urinary incontinence (OAB-wet).

Methods: The study included female OAB-wet patients aged 65 and above who were already taking OAB medication. Data obtained from electronic medical records were subjected to propensity score matching. All patients received instructions on pelvic floor exercises and were prescribed an appropriate dose of OAB medication. The VEL+UEL group (n=30) underwent three monthly laser sessions, while the control group (n=30) did not receive the treatment. Clinical outcomes were evaluated using the Overactive Bladder Symptom Score (OABSS), International Consultation on Incontinence Questionnaire-Short Form (ICIQ-SF), three-day urination diary, and Vaginal Health Index Score (VHIS). Medication usage and adverse events were also assessed. Statistical analysis and R code were performed using the AI chatbot GPT-4.0.

Results: The VEL+UEL group showed significant improvements in OABSS score, ICIQ-SF score, voided volume, daytime frequency, nocturia, and VHIS after 12 months of treatment (p<0.001). Notably, 13.3% of patients transitioned from OAB-wet to OAB-dry. In contrast, the control group did not exhibit significant changes. Medication use was significantly reduced in the VEL+UEL group compared to the control group (p<0.001). No long-term side effects were reported.

Conclusion: Combination therapy with VEL+UEL demonstrated efficacy and safety in the treatment of OAB-wet. Improvements in OAB symptoms, voided volume, frequency, nocturia, and vaginal health were observed, with a subset of patients transitioning to OAB-dry. VEL+UEL therapy offers a potential treatment option for OAB-wet, reducing medication use and improving patient outcomes. Further research is warranted to investigate the mechanism, long-term effects, safety, and cost-effectiveness of VEL+UEL therapy.

## Introduction

Overactive bladder (OAB) is characterized by a sense of urgency to urinate, accompanied by a frequency of urination and nocturia, regardless of the presence of urge urinary incontinence (UUI) [1]. Individuals with urinary incontinence are specifically referred to as having OAB-wet, which leads to a significant decline in their quality of life [2,3]. Urinary incontinence associated with OAB-wet can be categorized into different types, including UUI, stress urinary incontinence (SUI), or mixed urinary incontinence (MUI), combining SUI and UUI [4]. It has been observed that performing a mid-urethral sling (MUS) [5], which is a treatment for SUI, often worsens OAB in patients with MUI [6]. Therefore, the treatment of OAB-wet primarily relies on medications or injections that act on the bladder. As a result, in daily clinical practice, it is common to see cases where urinary incontinence is not completely resolved, and the use of OAB medications increases.

In recent years, the regenerative effects of laser therapy on cells have been shown to be effective for the treatment of urinary incontinence. In particular, non-ablative erbium:yttrium aluminum garnet (Er:YAG) laser treatment, which involves irradiation of the vagina and urethra, has demonstrated effectiveness not only for SUI but also for MUI. Initial reports focused on improving vaginal health in genitourinary syndrome of menopause (GSM) [7] and SUI [8] using a vaginal Er:YAG laser (VEL). Subsequently, in 2018, comparisons were made with MUS surgeries such as tension-free vaginal tape [9], revealing the absence of de novo UUI, and in 2019, it was tried for OAB treatment. In 2018, the urethral Er:YAG laser (UEL) technique was developed [10], and in 2023, VEL+UEL treatment was reported [11-13].

We assumed that the VEL+UEL treatment would be effective for OAB-wet. In this retrospective study, we investigated the effectiveness of this treatment in female patients who were already taking OAB medications.

## Materials and methods

Study design and approval

This was a single-center, retrospective, propensity score (PS)-matched cohort study conducted at Yokosuka Urogynecology and Urology Clinic, Kanagawa, Japan. The study was approved by the Ethical Review Board of Yokosuka Urogynecology and Urology Clinic (approval number: 24-C001). Data were extracted from the electronic medical records of the patients in our institution. All patients provided informed consent and signed the documents. The statistical analysis was performed at Kanagawa Dental University.

Patient recruitment and enrollment

In November 2016, a website was established to provide information on OAB, OAB medications, and laser treatments. The website promoted treatment options using keywords such as "PFMT", "frequency", "urinary incontinence" and "overactive bladder". As the number of patients visiting our clinic increased over the next three years, we were able to initiate the study as planned in 2019. From January 1, 2019, to December 31, 2021, a total of 625 patients (women aged ≥ 65 years) with symptoms of OAB and POP (stage 2 or below) were enrolled in the study. The patient breakdown by country was as follows: 615 Japanese, six Chinese, and four Korean. All were residents of Japan.

Data collection and interventions

Participants completed questionnaires including the Overactive Bladder Symptom Score (OABSS), International Consultation on Incontinence Questionnaire-Short Form (ICIQ-SF), a three-day urination diary, and underwent a pelvic organ prolapse (POP) examination conducted by a physician. All participants underwent pelvic floor muscle training (PFMT). Patients were divided into two groups based on their treatment: (i) the VEL+UEL group and (ii) the control group. Both groups received OAB medication for one year. The VEL+UEL group underwent a three-month VEL+UEL treatment in addition to PFMT, while the control group received PFMT alone. To minimize potential confounding factors, PS matching was performed to ensure comparability between the two groups in terms of the duration of OAB, OABSS, and ICIQ-SF scores at baseline. Patients were followed up every three months for 12 months.

Group characteristics and PS matching

The VEL+UEL group comprised 30 individuals who received three monthly sessions of treatment, and all of them successfully completed the one-year follow-up period without dropout. The control group initially comprised 333 cases, but within one year, 26 individuals dropped out, resulting in a final count of 307 cases. Reasons for dropout were either discontinued regular visits or failure to meet the medication adherence criteria (less than 70% adherence). After conducting propensity score matching based on the duration of OAB, OABSS, and ICIQ-SF score, finally, there were 30 individuals each in the VEL+UEL and control groups.

Eligibility criteria, study timeline, and follow-up

The eligibility criteria for VEL+UEL treatment were as follows: (i) patient's consent for the procedure, (ii) insufficient improvement in urinary incontinence with PFMT, (iii) no congenital abnormalities in the urogenital tract, (iv) absence of malignant tumors in the pelvic region, and (v) no history of urethral surgery. Exclusion criteria for the study were as follows: (i) participants opting out during the opt-out period, (ii) interruption of the one-year follow-up period, and (iii) presence of cognitive impairment or mental disorders that prevented completion of the questionnaire.

In this study, T0 was defined as the point at which OAB medication was initiated and six months had elapsed. The VEL+UEL group underwent the three-month VEL+UEL treatment after reviewing the data at T0. A one-year treatment record was maintained starting from one month after the third laser therapy. The control group was based on a one-year observational record from the three-month point (nine months after initiating OAB medication) at T0. For patients who had been receiving OAB treatment prior to the study, T0 was determined as the point at which we assessed the appropriateness of their medication dosage for a period of six months at our clinic [14]. All patients were evaluated every three months.

The pharmacist assessed the remaining medications during each follow-up visit. Medication use was continued for at least 12 months, and the patients were evaluated over the entire 12-month period (T12). The study included patients aged ≥ 65 years with American Society of Anesthesiologists Physical Status Class I to III. Patients with POP stage III or higher were prioritized for POP surgery rather than OAB treatment and were not included in this study [15].

Categorization of OAB

Two categories of OAB were determined.

OAB-Wet

Symptoms of frequency and urgency, frequency and nocturia, or frequency, urgency, and nocturia with UUI, SUI, or MUI combining SUI and UUI [16].

OAB-Dry

Symptoms of frequency and urgency, frequency, nocturia, frequency, urgency, and nocturia without urinary incontinence [16].

Method of evaluation

The OABSS questionnaire [17] evaluated OAB severity using four items: daytime frequency, nocturia, urgency, and incontinence. Scores ranged from 0 to 15, with scores <5 indicating mild OAB, 6-11 indicating moderate OAB, and ≥12 indicating severe OAB.

The OAB-wet and OAB-dry categories were based on patient self-reporting. If a patient with OAB-wet had an improvement in symptoms and transitioned to OAB-dry during the course of treatment, it was reported by the patient.

The ICIQ-SF questionnaire [18] assesses urinary incontinence in terms of frequency, amount, and impact on daily life. Scores ranged from 1 to 21 with 1-5 indicating mild incontinence, 6-12 indicating moderate incontinence, 13-18 indicating severe incontinence, and 19-21 indicating very severe incontinence.

From a three-day urination diary [19], the average daytime frequency, average nocturnal frequency, and average voided volume were calculated.

The Vaginal Health Index Score (VHIS) [20] assessed vaginal health based on elasticity, fluid secretion, pH, epithelial integrity, and moisture content. Each criterion was rated from 1 (very poor) to 5 (excellent). A total VHIS score < 15 indicated poor vaginal health.

PFMT

During appointments, patients were advised to perform daily 30-minute PFMT exercises. A nurse and doctor administered PFMT to guide the patients on vaginal muscle contractions. A video tutorial was shared for the home exercises. Exercise logs were reviewed, and encouragement was provided to the diligent patients.

OAB treatment drug

During the course of this study, the most commonly used OAB treatment drugs were fesoterodine fumarate (8 mg and 4 mg) [21] as an antimuscarinic drug and the β3 adrenoceptor agonist mirabegron (50 mg and 25 mg) [22]. According to the Japanese government guidelines, these medications can be administered concomitantly [23].

During the course of this study, Onabotulinumtoxin A bladder injections [24], sacral nerve stimulation [25], and posterior tibial nerve stimulation [26] were used. Urethral collagen injections were also restricted to overseas treatment because of the lack of approved usage in the country. Mid-urethral slings were not recommended for the treatment of MUI in patients with OAB. These drugs, adhering to the prescribed dosages recommended by the Japanese Pharmaceuticals and Medical Devices Agency, are covered by insurance.

Medication adherence to OAB treatment was confirmed by the pharmacist through assessment of the remaining medications returned to the pharmacy. For statistical analysis purposes, the OAB medication use rate was determined based on the criteria of medication usage ≥70% within the three months prior to the interview, irrespective of the specific dosage.

Medication use below 70% within the three-month period was considered nonadherence. Patients were categorized into two groups according to their physician's evaluation of the reasons for medication discontinuation. Group A comprised patients who no longer required OAB medication because of the potential effects of both the VEL+UEL treatment and PFMT. If the usage fell below 50% by T12, it was considered unnecessary. The analysis focused on the rate of OAB medication reduction within this group, taking the therapeutic effects into account. Group B consisted of patients who either forgot or chose not to take the medication because of side effects. Patients who participated in the study for less than one year were excluded from the analysis.

VEL+UEL treatment

The VEL+UEL procedures were performed consecutively. Both steps utilized the non-ablative Er:YAG SMOOTH laser mode (SP Dynamis; Fotona d.o.o., Ljubljana, Slovenia). In the VEL step, 8% xylocaine spray (Sandoz Group AG, Basel, Switzerland) was applied. No sedatives were administered. A vaginal probe and a glass vaginal speculum were inserted into the vagina. The anterior vaginal wall was treated using a PS03 laser probe with a frequency of 2.0 Hz, pulse fluence of 6 J/cm^2^, and spot size of 7 mm. The target area was repeatedly irradiated, with each irradiation being spaced 5 mm apart. In the subsequent step, the R11 laser probe was used to provide 360-degree coverage of the entire vaginal canal. The pulse parameters included a frequency of 2.0 Hz, a spot size of 7 mm, and a pulse fluence of 3.00 J/cm^2^. Laser irradiation was applied every 5 mm along the vaginal canal and the procedure was repeated twice. This process was completed within approximately 20 minutes. In the UEL step, the same laser model was employed. The R09-2Gu laser probe was inserted into the urethra after bladder emptying through catheterization. Laser therapy was performed using the following parameters: R09-2Gu, SMOOTH, 1.4 Hz, 1.5 J/cm^2^, with four stacks at 2.5 mm intervals from the urethral opening to the proximal end. This treatment was repeated four times and concluded within approximately 10 minutes. The patient was advised to refrain from sexual intercourse and masturbation for one week following treatment [11].

Statistical analysis

All analyses were conducted using the data presented to the artificial intelligence chatbot GPT-4.0 (OpenAI, San Francisco, California, United States), which proposed statistical methods and generated R code. R version 2.15.1 (R Core Team, Vienna, Austria) and the EZR package for the Windows 10 operating system version 1903 (Microsoft Corporation, Redmond, Washington, United States) were utilized. Fisher’s exact test was used for between-group comparisons of discrete variables. Summary statistics were presented as means (standard deviations) or numbers (percentages). The Student's t-test was used to compare continuous variables between the two groups. Statistical significance was set at p < 0.05. The differences in the parameters between T0 and T12 (ΔT0/T12) were calculated by subtracting T0 from T12.

## Results

**Figure 1:**
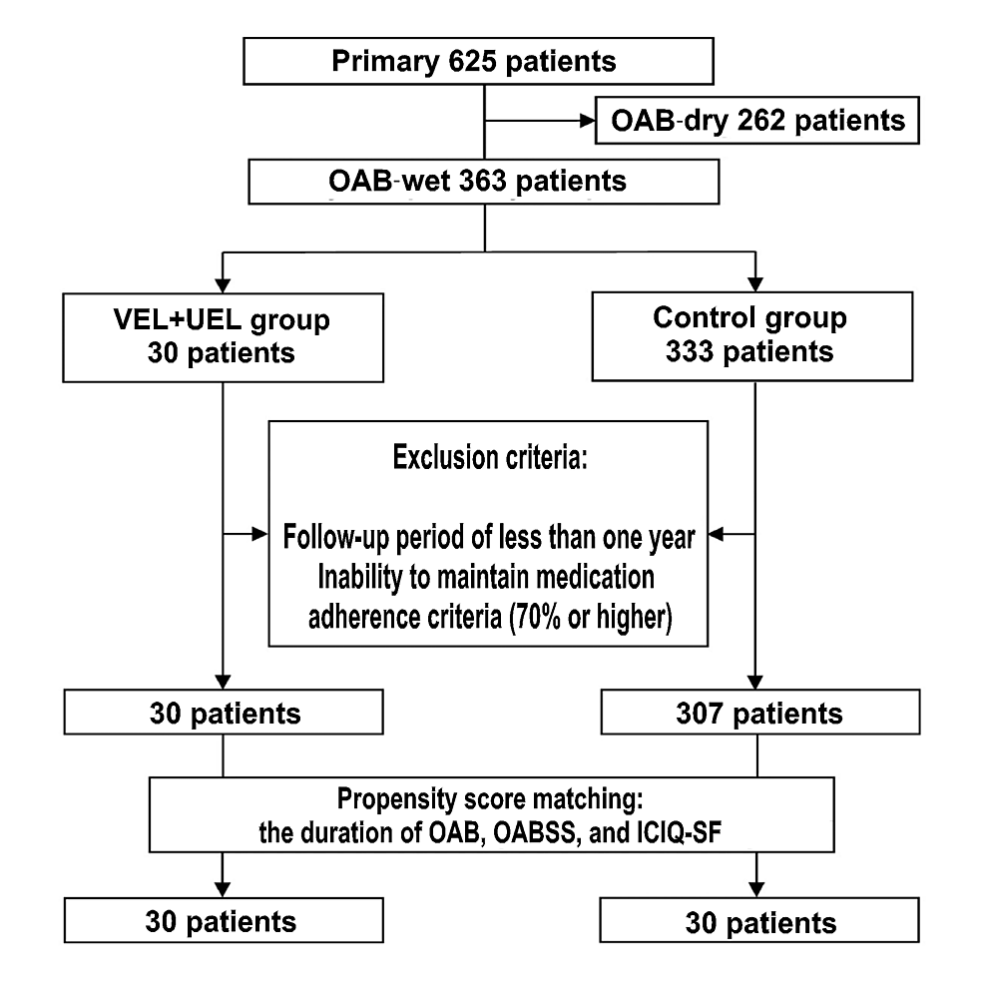
Study flowchart showing the inclusion and exclusion criteria OAB: overactive bladder; OAB-dry: overactive bladder without urinary incontinence; OAB-wet: overactive bladder with urinary incontinence; VEL: vaginal erbium:yttrium aluminum garnet laser; UEL: urethral erbium:yttrium aluminum garnet laser; ICIQ-SF: International Consultation on Incontinence Questionnaire-Short Form; OABSS: Overactive bladder symptom score

The VEL+UEL group comprised 30 individuals who received three monthly sessions of treatment, and all of them successfully completed the one-year follow-up period without dropout. None of the patients in the VEL+UEL group discontinued OAB medication solely because of the effects of PFMT. The control group initially comprised 333 cases, but 26 individuals dropped out, resulting in 307 cases as shown in Figure 1. After conducting PS matching based on the duration of OAB, OABSS, and ICIQ-SF score, there were 30 individuals each in the VEL+UEL and control groups.

Table 1 shows the comparison results after PS between the VEL+UEL group (n=30) and the control group (n=30). The comparison focused on patient background factors such as age and BMI, with statistical significance indicated by p-values. In this comparison, no significant differences were observed between the two groups in factors such as age (p = 0.625) and BMI (p = 0.878). Similarly, there were no significant differences between the groups for factors such as alcohol consumption, smoking, hypertension, hyperlipidemia, and diabetes (p > 0.05).

**Table 1:** Comparison between the VEL+UEL and control groups at T0 after propensity score VEL: vaginal erbium:yttrium aluminum garnet laser; UEL: urethral erbium:yttrium aluminum garnet laser

Characteristics	VEL+UEL group (n=30)	Control group (n=30)	p-value
Age (years), mean±SD	69.26 ± 3.16	68.00 ± 3.46	0.625
BMI (*kg/m^2^)*, mean±SD	23.01 ± 1.81	23.81 ± 2.96	0.878
Alcohol (> 3 days a week), n (%)	2 (6.6%)	2 (6.6%)	1.00
Smoking, n (%)	2 (6.6%)	2 (6.6%)	1.000
Hypertension, n (%)	10 (33.3%)	12 (40.0%)	0.724
Hyperlipidemia, n (%)	10 (33.3%)	12 (40.0%)	0.724
Diabetes, n (%)	5 (16.7%)	4 (13.3%)	0.623
Number of pregnancies, mean±SD	2.23 ± 0.62	2.20 ± 0.65	0.751
Number of vaginal deliveries, mean±SD	2.1 ± 0.539	2.1 ± 0.597	0.581
Bladder cancer, n (%)	0 (0%)	0 (0%)	0
Bladder stone, n (%)	0 (0%)	0 (0%)	0
Surgery for pelvic organ prolapse, n (%)	0 (0%)	0 (0%)	0
Uterine fibroids (>10 cm in diameter), n (%)	0 (0%)	0 (0%)	0
Uterine fibroids (> 3 cm in diameter), n (%)	2 (6.6%)	2 (6.6%)	1.000
History of endometriosis, n (%)	2 (6.6%)	2 (6.6%)	1.000
Ovarian cyst, n (%)	6 (20%)	3 (10%)	0.153

Table 2 shows the results of the comparison between the two groups after PS matching in the field of urogynecology. At T0, there were no statistically significant differences between the two groups in terms of the duration of OAB affliction (p = 0.893), OABSS (p = 0.823), ICIQ-SF score (p = 0.893), daytime frequency (p = 0.758), nocturia (p = 0.292), voided volume (p = 0.538), or VHIS (p = 0.488).

**Table 2:** Comparison between the VEL+UEL and cpntrol groups at T0 in the field of urogynecology after propensity score OAB: overactive bladder; ICIQ-SF: International Consultation on Incontinence Questionnaire-Short Form; OABSS: overactive bladder symptom score; VHIS: Vaginal Health Index Score; VEL: vaginal erbium:yttrium aluminum garnet laser; UEL: urethral erbium:yttrium aluminum garnet laser

Measurement scores	VEL+UEL group (n=30), mean±SD	Control group (n=30), mean±SD	p-values
Duration of OAB affliction (years)	2.86 ± 1.20	2.93 ± 1.17	0.893
OABSS	7.33 ± 1.08	7.52 ± 1.13	0.823
ICIQ-SF	10.86 ± 0.88	10.90 ± 0.89	0.893
Daytime frequency	10.5 ± 1.43	10.62 ± 1.34	0.758
Nocturia	3.06 ± 0.68	2.96 ± 0.55	0.292
Voided volume (ml)	142.6 ± 24.8	145.9 ± 21.6	0.538
VHIS	12.5 ± 2.57	12.3 ± 2.94	0.488

Four patients (13.3%) in the VEL+UEL group showed an improvement from OAB-wet to OAB-dry. However, no significant differences were observed in the control group.

**Figure 2:**
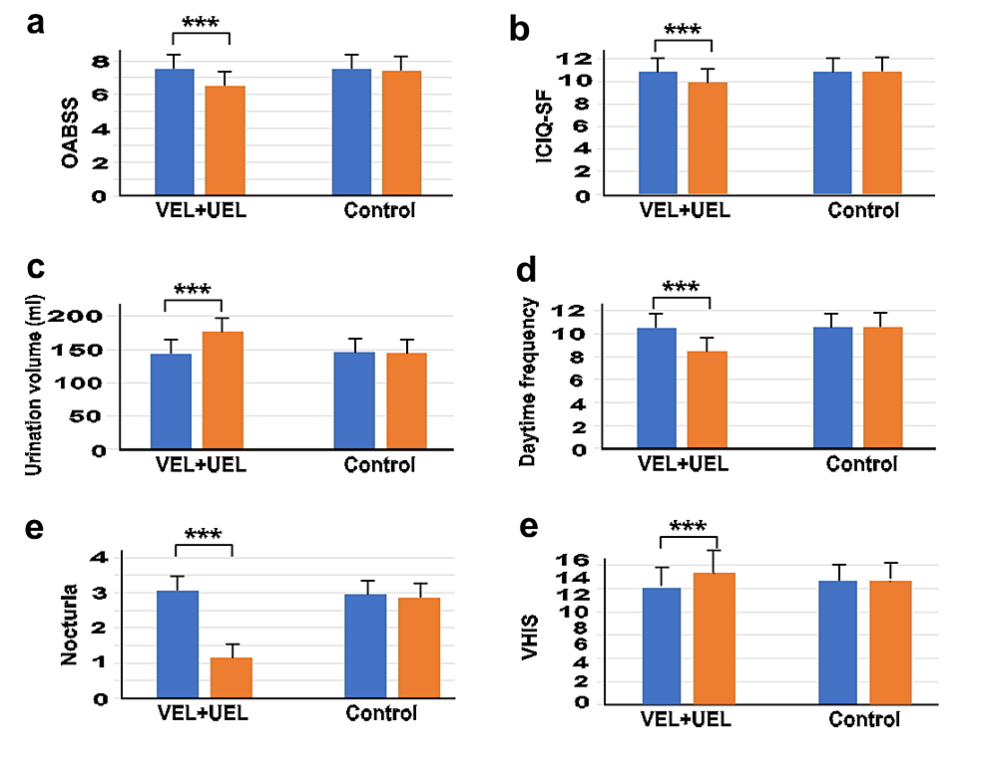
Comparison of OABSS, ICIQ-SF score, urination volume, daytime frequency, nocturia, and VHIS between the two groups at T0 and T12 Vertical axis: (a) OABSS, (b) ICIQ-SF score, (c) urination volume (ml), (d) daytime frequency, (e) nocturia, and (f) VHIS; Horizontal axis: VEL+UEL group and Control group at T0 (blue bar) and T12 (orange bar). T0 represents the data before treatment and T12 represents the data after 12 months of treatment. *** indicates significant differences with p-value < 0.001 PS: propensity score; OAB: overactive bladder; ICIQ-SF: International Consultation on Incontinence Questionnaire-Short Form; OABSS: overactive bladder symptom score; VHIS: Vaginal Health Index; VEL: vaginal erbium:yttrium aluminum garnet laser; UEL: urethral erbium:yttrium aluminum garnet laser

Figure 2 shows the changes in OABSS, ICIQ-SF, urination volume, daytime frequency, nocturia, and VHIS scores for each group. Significant differences were observed between T0 and T12 in all parameters in the VEL+UEL group. Conversely, there were no significant differences between T0 and T12 in any of the parameters in the control group. The ΔT0/T12 OABSS scale score showed a significantly higher decrease in the VEL+UEL group (-2.4 ± 2.22) than in the control group (-0.10 ± 0.48) (p<0.001). Similarly, the ΔT0/T12 ICIQ-SF score exhibited a significantly higher decrease in the VEL+UEL group (-2.1 ± 3.11) than in the control group (-0.035 ± 0.18) (p<0.001). In terms of urinary parameters, the ΔT0/T12 urination volume showed a significantly higher increase in the VEL+UEL group (34.9 ± 9.61) than in the control group (-0.965 ± 3.56) (p<0.001). Additionally, the ΔT0/T12 daytime frequency and nocturia demonstrated significantly higher decreases in the VEL+UEL group (-2.27 ± 0.51 and -1.9 ± 0.94, respectively) compared to the control group (-0.03 ± 0.41 and -0.10 ± 0.40, respectively) (p<0.001). Furthermore, the ΔT0/T12 VHIS showed a significantly higher increase in the VEL+UEL group (1.8 ± 1.86) than in the control group (0.37 ± 2.16) (p<0.001).

**Figure 3:**
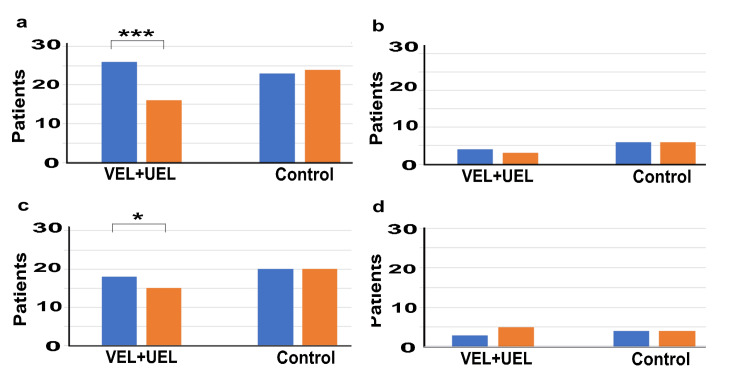
Oral OAB medication use rate in the two groups at T0 and T12 Vertical axis: Number of patients using oral drugs; Horizontal axis: VEL+UEL group and Control group at T0 (blue bar) and T12 (orange bar): (a) Fesoterodine fumarate 8 mg, (b) Fesoterodine fumarate 4 mg, (c) Mirabegron 5 mg, (d) Mirabegron 2.5 mg T0 represents the data before treatment, and T12 represents the data after 12 months of treatment. *** indicates significant differences with p-value < 0.001; * indicates significant differences with p-value < 0.05 OAB: overactive bladder; VEL: vaginal erbium:yttrium aluminum garnet laser; UEL: urethral erbium:yttrium aluminum garnet laser

Figure 3 shows the OAB medication use in the two groups after PS matching. At T0, in the VEL+UEL group, 26 individuals (86.6%) were taking fesoterodine fumarate at a dose of 8 mg, and four individuals (13.3%) were taking it at a dose of 4 mg. However, at T12, these numbers decreased to 16 (53.3 %) and three (10 %) individuals, respectively. In contrast, in the control group, 23 individuals (76.6%) were taking fesoterodine fumarate at a dose of 8 mg, and six individuals (20%) were taking it at a dose of 4 mg at T0. At T12, these numbers changed to 24 individuals (80%) and six individuals (20%), respectively. At T0, in the VEL+UEL group, 18 individuals (60%) were taking mirabegron at a dose of 50 mg, and three individuals (10%) were taking it at a dose of 25 mg. However, at T12, these numbers changed to 15 (50 %) and five (16.6 %) individuals, respectively. In contrast, in the control group, 20 individuals (66.7%) were taking mirabegron at a dose of 50 mg and four individuals (13.3%) were taking mirabegron at a dose of 25 mg at T0. There were no changes in numbers at T12.

**Figure 4:**
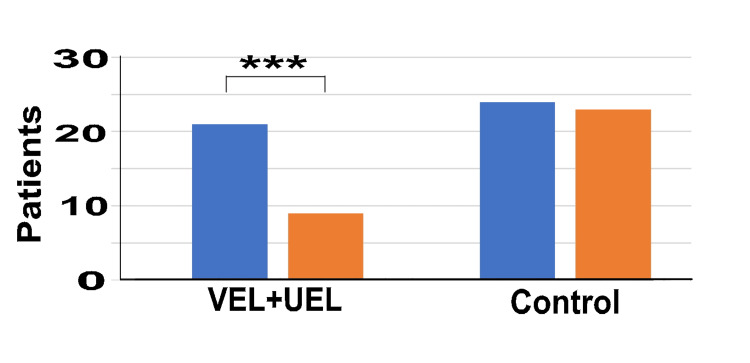
Number of patients using two types of oral OAB medication simultaneously at T0 and T12 Vertical axis: Number of patients using oral drugs; Horizontal axis: VEL+UEL group and Control group at T0 (blue bar) and T12 (orange bar) T0 represents the data before treatment, and T12 represents the data after 12 months of treatment. *** indicates significant differences with p-value < 0.001 OAB: overactive bladder; VEL: vaginal erbium:yttrium aluminum garnet laser; UEL: urethral erbium:yttrium aluminum garnet laser

Figure 4 shows the number of patients using the two types of oral OAB medications in the two groups after PS matching. At T0, in the VEL+UEL group, 21 individuals (70%) received concomitant administration of fesoterodine fumarate and mirabegron, while in the control group, 24 individuals (80%) received the same combination. However, at T12, the number of individuals receiving concomitant administration in the VEL+UEL group decreased to nine (30%), whereas in the control group, it remained at 23 individuals (76.7%).

After VEL+UEL therapy, two patients experienced pain during treatment. In addition, one patient experienced mild cystitis after treatment. However, no long-term side effects were reported.

## Discussion

We conducted a retrospective PS-matching cohort study to evaluate the clinical efficacy and safety of VEL+UEL therapy in patients with OAB-wet. The results showed that the VEL+UEL group exhibited significant improvements in the OABSS score, ICIQ-SF score, voided volume, daytime frequency, nocturia, and VHIS from T0 to T12. In contrast, the control group showed no significant changes in these parameters.

While our study is the first, according to the best of our knowledge, to investigate the combination of VEL+UEL therapy for OAB-wet treatment, there have been several studies on VEL monotherapy for OAB. One of the initial studies by Okui, conducted in 2019, compared VEL with anticholinergic medication (fesoterodine fumarate 4 mg) and a β3-adrenergic receptor agonist (mirabegron 25 mg) [27]. The study measured OABSS and VHIS and reported improvements in OABSS scores from 8.16 ± 2.86 to 3.76 ± 3.30 for the VEL group, from 7.96 ± 2.49 to 4.16 ± 2.59 for the anticholinergic group, and from 8.30 ± 2.88 to 5.25 ± 3.08 for the β3 group. Although the study targeted treatment-naïve individuals and differed from our research, it suggests a similar trend.

Furthermore, a study by Gambacciani and Okui, that focused on patients already receiving OAB medication, compared four treatment groups: control, VEL, mirabegron 25 mg, and mirabegron 25 mg+VEL [28]. The VEL group, mirabegron group, and mirabegron + VEL group all showed improvements in OABSS scores from 8.00 ± 2.38 to 4.85 ± 2.38, from 8.00 ± 2.73 to 5.80 ± 3.22, and from 7.95 ± 2.41 to 4.25 ± 2.02, respectively, while the control group exhibited no changes. In particular, both the VEL and mirabegron 25 mg + VEL groups demonstrated similar strong effects on nocturia, suggesting that VEL and mirabegron 25 mg have different mechanisms of action. This study indicates the potential of combining VEL with OAB medications, which is similar to our findings.

Additionally, a study conducted by Chiengthong et al. in 2023 involved a single-center randomized placebo-controlled trial with a 12-week investigation period using the Thai version of the Overactive Bladder questionnaire (OAB-q) [29]. The VEL group and sham group showed significant improvements in the total score (6.03 ± 3.36 vs 8.44 ± 3.39, P = 0.015) and nocturia (1.71 ± 0.74 vs 2.32 ± 0.70, P = 0.004). Unlike our study, their study divided subjects into groups that either received one session of VEL treatment or underwent a sham procedure without prior OAB treatment. Honma et al. suggest a relatively close correlation between OABSS and OAB-q [30]. Similar to our use of the OABSS, the effectiveness of VEL treatment in reducing nocturia is noteworthy. Nocturia has long been a challenge with OAB medications [21].

Comparing these previous studies with our research findings, VEL+UEL therapy appears to be effective in OAB patients and has the potential to contribute to symptom improvement. Specifically, improvements were observed in the OABSS, ICIQ-SF, voided volume, daytime frequency, nighttime frequency, and VHIS. These results suggest that VEL+UEL therapy may have a comprehensive therapeutic effect on various aspects of OAB.

Of particular note is the potential to reduce the dosage of OAB medications. In addition to the medications used in our study, such as vibegron, which ranks among the top globally used drugs in 2023 [31], there are options, such as onabotulinumtoxin A bladder injections and nerve stimulation, when medication therapy is ineffective. However, these methods do not aim to regenerate the cells in the bladder or urethra. In the case of VEL+UEL therapy, as demonstrated in Okui et al. [6] and Okui [7], and in the current study, improvements in vaginal health status have been observed. Furthermore, case reports of VEL+UEL therapy have shown that the morphology of the urethra becomes rounder, and the muscles become more balanced [11]. Based on the above evidence, VEL+UEL therapy has the potential to serve a supportive role in current OAB treatments through a different mechanism.

Furthermore, it is important to consider the role of PFMT in VEL+UEL therapy. In our study, all patients received instructions on PFMT and were encouraged to perform daily exercise. PFMT has been established as a conservative treatment for neurogenic bladder dysfunction in women after spinal cord injury [32]. This can help improve pelvic floor muscle strength and coordination, leading to better bladder control. Although our study did not focus on neurogenic bladder dysfunction, the combination of PFMT and VEL+UEL therapy may have contributed to the observed improvements in OAB symptoms and quality of life. Future studies should investigate the specific effects of PFMT in conjunction with VEL+UEL therapy, and explore the optimal protocol for combining these treatments in various patient populations, including those with neurogenic bladder dysfunction.

However, our study had several limitations. First, as this was a retrospective PS-matched cohort study, the observed effects and side effects may be influenced by other factors. Additionally, the study duration was relatively short, limiting the evaluation of the long-term effects and safety. Furthermore, the study population was limited to a specific age group (women aged ≥ 65 years), necessitating further investigation of its applicability to the general population.

Future studies should include larger randomized comparative trials and long-term follow-up. It is also important to consider the mechanism of action of VEL+UEL therapy, compare it with other treatment methods, evaluate its safety, and consider its economic aspects.

## Conclusions

Our study supports the efficacy and safety of the VEL+UEL treatment for OAB-wet. Significant improvements in the OAB symptoms, voided volume, frequency, nocturia, and vaginal health index scores were observed after 12 months of treatment. At least 13.3% of patients transitioned from OAB-wet to OAB-dry. VEL+UEL treatment has the potential to improve OAB symptoms and quality of life. Additionally, no significant side effects were observed with VEL + UEL treatment.

Our study suggests that VEL+UEL treatment may offer a safe and effective option for managing OAB-wet. Further research is needed to explore the mechanism, safety, and economic aspects of VEL+UEL treatment through larger trials and long-term follow-up, contributing to improved patient outcomes.
